# Differential roles of insulin like growth factor 1 receptor and insulin receptor during embryonic heart development

**DOI:** 10.1186/s12861-019-0186-8

**Published:** 2019-03-25

**Authors:** Kai Wang, Hua Shen, Peiheng Gan, Susana Cavallero, S. Ram Kumar, Ching-Ling Lien, Henry M. Sucov

**Affiliations:** 1 Department of Cardiovascular Surgery, the First Affiliated Hospital of Guangzhou Medical University, Guangzhou, 510120 China; 2 Department of Stem Cell Biology and Regenerative Medicine, Keck School of Medicine, University of Southern California, Los Angeles, CA USA; 3 Department of Surgery, Keck School of Medicine, University of Southern California, Los Angeles, CA USA; 4 Saban Research Institute, Children’s Hospital Los Angeles, Los Angeles, CA USA; 5 Department of Regenerative Medicine and Cell Biology, Medical University of South Carolina, Charleston, SC USA; 6 Department of Medicine, Division of Cardiology, Medical University of South Carolina, Charleston, SC USA

**Keywords:** Heart development, Epicardium, IGF2, IGF1R, Insulin receptor, Cardiomyocyte proliferation

## Abstract

**Background:**

The embryonic day E10–13 period of mouse heart development is characterized by robust cardiomyocyte proliferation that creates the compact zone of thickened ventricular wall myocardium. This process is initiated by the formation of the epicardium on the outer heart surface, which releases insulin-like growth factor 2 (IGF2) as the primary cardiomyocyte mitogen. Two receptors mediate IGF2 signaling, the IGF1R and the insulin receptor (INSR).

**Results:**

In this study, we addressed the relative roles of the two IGF2 receptors in mouse heart development. We find that both receptors are expressed in the mouse heart during the E10–13 period, although IGF1R is much more prominently activated by IGF2 than INSR. Genetic manipulation indicates that only *Igf1r* is required for embryonic ventricular wall morphogenesis. INSR is not hyperactivated in the absence of IGF1R, and INSR does not compensate functionally for IGF1R in the absence of the latter.

**Conclusions:**

These results define the molecular components that are responsible for a major burst of cardiomyocyte proliferation during heart development. These results may also be relevant to understanding the efficiency of regeneration of the mammalian heart after neonatal and adult injury.

**Electronic supplementary material:**

The online version of this article (10.1186/s12861-019-0186-8) contains supplementary material, which is available to authorized users.

## Background

The heart ventricular wall is responsible for most of the contractile force of each heartbeat [1]. In heart development, ventricular wall morphogenesis can be divided into several discrete stages, including its initial formation from mesodermal progenitors (in mouse, from embryonic day E7–10), a rapid midgestation expansion (E10–13) based on cardiomyocyte proliferation, and a later phase of declining rate of growth as the final number of cardiomyocytes is reached (E14-term). The E10–13 midgestation period of growth not only achieves an increase in cardiomyocyte cell number, but also is morphologically organized to increase ventricular wall thickness, which is more mechanically supportive of stronger cardiac contraction [1]. Previous observations indicated that initiation of this midgestation period of ventricular wall expansion at E10 is coincident with the formation of the epicardium, which migrates onto the outer heart surface from a nearby origin and rapidly spreads into a single cell layer of mesothelium [2], and furthermore that the epicardium is in fact required for this phase of cardiomyocyte proliferation [3]. We previously showed that the epicardium is the source of secreted mitogenic factors [4, 5], and then demonstrated that insulin-like growth factor 2 (IGF2) is the primary epicardial mitogen responsible for E10–13 ventricular wall cardiomyocyte proliferation and morphogenesis [6]. *Igf2* mRNA is expressed in the epicardium and in the endocardium (the inner endothelium of the heart), but not in the myocardium. A component of our subsequent analysis was conditional mutation of the *Igf2* gene in all heart mesoderm (using *Nkx2.5Cre*), or specifically in the epicardium (using *Tbx18Cre*), both of which resulted in a hypoplastic ventricular wall and a deficiency of cardiomyocyte proliferation [7]. Conditional mutation of *Igf2* in the endocardium-endothelium lineage (using *Tie2Cre*) had no phenotypic consequence [7].

IGF2 signaling can be mediated by two receptors of the transmembrane receptor tyrosine kinase family, the insulin receptor (INSR) and the insulin-like growth factor 1 receptor (IGF1R). Each receptor also has nonoverlapping specificity for insulin and IGF1, respectively [8]. Upon binding ligand, these receptors undergo autophosphorylation as the first step in initiating intracellular signaling. Structurally, IGF1R and INSR share approximately 70% homology, which allows them to function similarly in many tissues and in many regulatory pathways [9]. IGF1R is mostly considered as a typical growth factor receptor mediating mitogenic response and cell proliferation. INSR in the adult is mostly considered to mediate metabolic regulation in response to insulin, but in the embryo, where insulin levels are minimal, it clearly also has mitogenic and growth activity in response to IGF2 [10].

Because IGF2 response can be mediated by both IGF1R and INSR, in our previous study [6] we conditionally ablated both receptors together and observed the same phenotypic consequence as IGF2 ligand gene mutation. An unresolved question was whether the two receptors contribute equally, redundantly, or uniquely in this process. In this study, we demonstrate that both receptors are expressed, but show by biochemical and genetic approaches that IGF1R is the predominant IGF2 receptor in the developing heart.

## Results

### Expression and activation of IGF1R and INSR

**Fig. 1:**
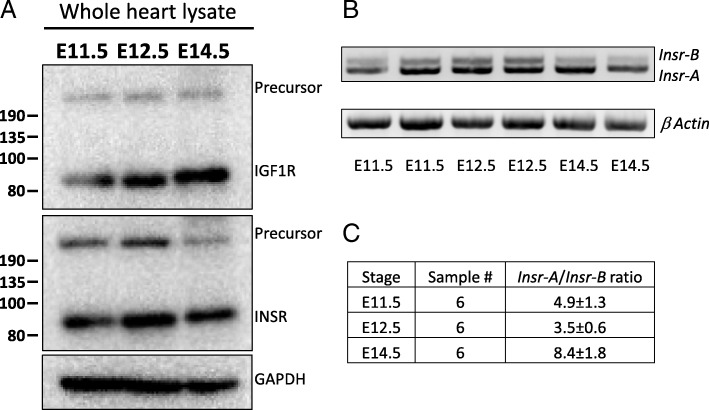
IGF1R and INSR in midgestation mouse heart. **a** Western blot analysis of IGF1R and INSR protein at the indicated developmental time points. The mature receptor proteins and the larger precursors prior to proteolytic cleavage are both visualized. **b** PCR amplification of *Insr* mRNA to detect the A and B transcript isoforms. **c** Quantitation of the *Insr-A/Insr-B* ratio based on amplification such as shown in panel B; value is mean ± SEM

In our previous study [6], we detected mRNA transcripts for *Igf1r* and *Insr* in the developing mouse heart ventricle by PCR amplification, but the level of message was too low to detect by in situ hybridization. We first confirmed protein expression by Western blotting, using whole heart tissue from embryos at E11.5, E12.5 and E14.5 (Fig. 1a). Both receptors were detectable at all time points. Based on the relative intensity of signal in the Western blots, it was not evident that the expression level of the two receptors was dramatically different from each other, although this assessment was not standardized in order to reach a quantitative conclusion, and also does not take into account potential regional differences in expression in the embryonic heart.

Two transcripts are produced from the *Insr* gene by alternative splicing of exon 11, resulting in the inclusion or exclusion of a 36 nt sequence. The resulting transcripts thereby encode proteins that differ by 12 amino acids in an extracellular domain of the receptor. The shorter isoform, called INSR-A, binds to and responds to both insulin and IGF2 with an affinity for IGF2 close to that of insulin, whereas the longer form, INSR-B, is selective for insulin [11]. Neither INSR isoform has significant affinity for IGF1 [11]. We detected both *Insr* transcripts in embryonic ventricle cDNA by PCR amplification (Fig. 1b), using primers from the common exons flanking exon 11, such that the relative level of each transcript can be quantitatively compared to the other in each sample. Throughout the E11.5–14.5 period, the *Insr-A* mRNA isoform of the insulin receptor gene was present at a level several-fold higher than *Insr-B* (Fig. 1c). Because the two transcripts differ only in one internal exon, it is unlikely that they are differentially translated, and the two proteins have a similar stability when expressed in transfected cells [12]. Thus, the INSR protein detected by Western blot (Fig. 1a) is most likely predominantly INSR-A, which is the IGF2-responsive isoform of INSR.

**Fig. 2:**
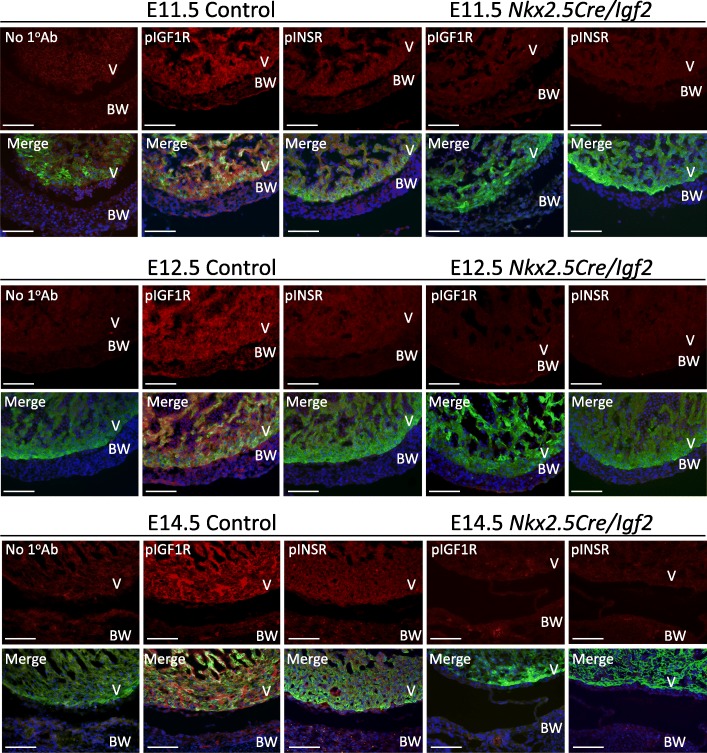
Immunofluorescence detection of activated (phosphorylated) receptors. Sections through the ventricular wall of control and conditional *Igf2* mutant embryos at E11.5, E12.5, and E14.5 are shown. Phospho-specific primary antibodies against pIGF1R and pINSR were used as indicated, or no primary antibody was used as a negative control for background. Pairs of images show the phospho-receptor staining in the red channel alone (top of each pair), or merged (lower of each pair) with the signal for a cardiomyocyte marker (see Methods; green channel) and for DNA (DAPI, blue channel). Abbreviations: BW, body wall; V, ventricle. Scale bar: 100 μm

For members of the receptor tyrosine kinase family, binding of ligand induces receptor autophosphorylation as the initial step of signal transduction. Therefore, the presence of phosphorylated receptor is an indication of active signaling. In control embryos, immunofluorescence using phospho-receptor-specific antibodies demonstrated that both receptors were active in the ventricle at E11.5, E12.5 and E14.5 (Fig. 2 left side, Additional file 1: Figure S1). Signal for phospho-IGF1R was readily visible in trabecular and compact (ventricular wall) myocardium, as well as in endocardium and coronary blood vessel endothelium. Phospho-INSR signal was detectable in a similar broad pattern but was much weaker.

We conducted a parallel immunofluorescence analysis of receptor phosphorylation in conditional *Nkx2.5Cre/Igf2* ligand gene mutant embryos (Fig. 2 right side). Importantly, for both receptors and at all three stages, signal was completely abolished in the absence of IGF2 ligand. This indicates that activation (phosphorylation) of the two receptors in the heart during midgestation is a consequence only of IGF2 signaling.

### Predominant function of IGF1R in heart ventricular development

**Fig. 3:**
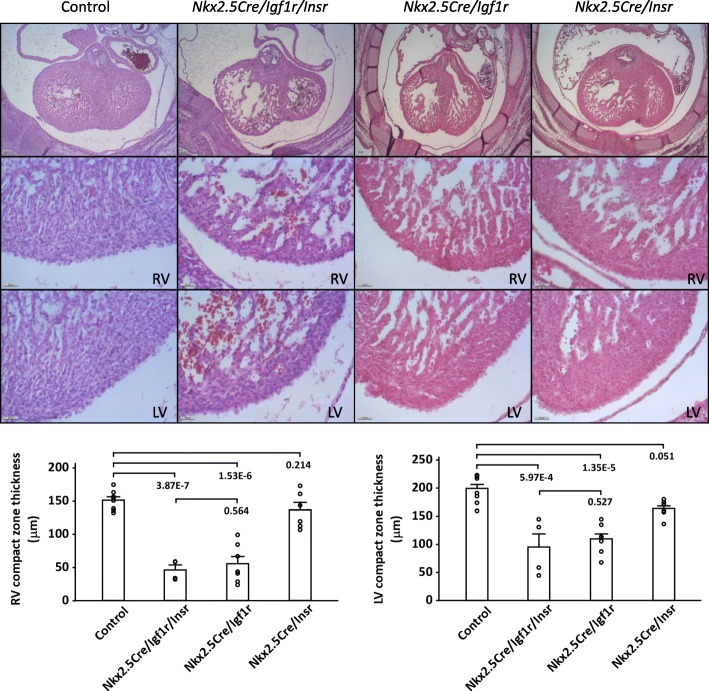
Phenotypic comparison of ventricular morphology in E14.5 control and conditional double and single receptor mutants. The conditional double mutants shown were newly generated for this study, but repeat a previously conducted analysis (ref. [6]). Scale bars (lower left corner of each panel) are 100 μm for low magnification panels (top row) and 50 μm for high magnification panels (middle and bottom rows). Quantitation of right and left ventricular wall thickness (mean ± SEM) is shown in the bar charts below. 8 normal embryos (from 4 litters) were used as the control. The numbers of conditional mutant embryos examined were as follows: *Nkx2.5Cre/Igf1r/Insr* (4 from 2 litters); *Nkx2.5Cre/Igf1r* (7 from 3 litters); *Nkx2.5Cre/Insr* (6 from 3 litters). Abbreviations: LV, left ventricle; RV, right ventricle

In our previous study, we evaluated embryos in which the *Igf1r* and *Insr* genes were conditionally mutated together. For this purpose, we used *Nkx2.5Cre*, which is active with high efficiency in all heart mesoderm, and *MLC2vCre*, which is cardiomyocyte-specific although not fully efficient. We observed a prominent thinning of the midgestation ventricular wall, more severe when using *Nkx2.5Cre*, and demonstrated this to be the consequence of reduced cardiomyocyte proliferation [6]. To address the relative contribution of IGF1R and INSR to this phenotype, we conditionally knocked out each receptor gene individually with *Nkx2.5Cre*, and compared to the double receptor mutant phenotype. *Igf1r* receptor gene mutation recapitulated the hypoplastic ventricular wall phenotype observed after combined double receptor mutation, in both the left and right ventricles (Fig. 3, Additional file 2). In contrast, *Insr* receptor gene knockout had at most only a minimal impact on ventricular wall formation. This outcome, along with the results shown in Fig. 2, indicate that IGF1R is the predominant receptor that regulates midgestation ventricular wall formation in response to IGF2. Although we did not measure proliferation in the present study, our past analysis of compromised cardiomyocyte proliferation in conditional *Igf2* ligand mutants and in conditional *Igf1r/Insr* double receptor mutants [6] implies that impaired cardiomyocyte proliferation is also the explanation for the thin ventricular wall phenotype in conditional *Igf1r* single gene mutants.

**Fig. 4:**
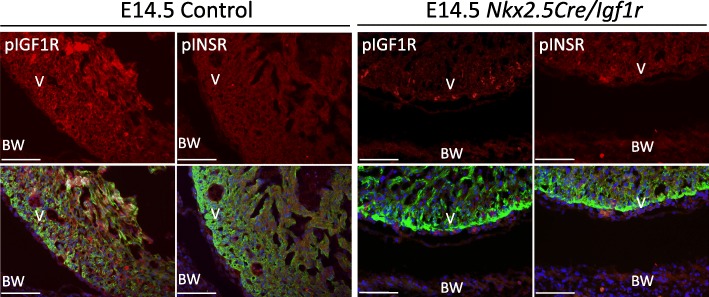
Lack of compensatory activation of INSR when IGF1R is absent. Immunofluorescence detection of pINSR and pIGF1R in E14.5 control and conditional *Igf1r* mutants. Analysis, image presentation (single channel and merged channels), and abbreviations are as in the legend to Fig. 2. Scale bar: 50 μm

We also evaluated the phosphorylation (activation) status of both receptors when *Igf1r* was conditionally mutated in the heart with *Nkx2.5Cre* (Fig. 4). As expected, phospho-IGF1R staining in the myocardium was eliminated by this genetic manipulation. Some residual positive signal was detected in endocardium, which is less efficiently recombined by *Nkx2.5Cre*, and in coronary endothelium that is not subject to recombination by *Nkx2.5Cre* [13]. Because IGF1R and INSR can both serve as receptors for IGF2, we considered whether *Igf1r* conditional mutation would change INSR phosphorylation status. However, phospho-INSR immunofluorescence signal was unchanged in conditional *Nkx2.5Cre/Igf1r* mutants (Fig. 4). This indicates that activation of INSR is not increased in a compensatory manner when IGF1R, the primary IGF2 receptor, is absent, and is consistent with the occurrence of the full ventricular hypoplastic phenotype as seen in double receptor mutants when only *Igf1r* is mutated in the midgestation heart.

## Discussion

Early heart morphogenesis, in mouse between E7–10, involves de novo derivation of new cardiomyocytes from progenitors and proliferation of these newly formed cardiomyocytes. Both processes together are organized such that the heart lengthens but remains thin-walled (1–3 cell diameters). The E10–13 period of mouse heart development represents a profound change in both logic and mechanism of ventricle wall morphogenesis. During this interval, the ventricular wall expands both radially as well as longitudinally, reaching approx. 15 cell diameters by E14. This phase of heart growth is instructed at least in part by the epicardium, which newly forms on the outer heart surface at E10, and which secretes mitogenic factors that stimulate cardiomyocyte proliferation. The importance of this process is evidenced by the large number of mouse mutations that have normal hearts at E10 and yet fail to accomplish ventricular wall expansion, resulting in a persistently thin-walled ventricle at E14 that is often so severe as to cause embryo lethality [14].

Our previous results [6, 7] defined the role of IGF2, and more generally the role of the epicardium as the source of secreted mitogens (primarily IGF2), in mouse midgestation heart development. IGF2 is relatively small (8kD), which presumably allows it to diffuse from the epicardium through the complete ventricular wall at E10. As we previously demonstrated [6], the genetic function of *Igf2* in ventricular wall cardiomyocyte proliferation is constrained to the E10–13 period, even though *Igf2* continues to be expressed in the epicardium (and endocardium) through the remainder of gestation [7]. One teleological explanation for the cessation of genetic relevance is that a diffusible mitogen that originates from the epicardium and is therefore limited to the outer heart surface would have progressively less impact as the thickness of the ventricular wall increases by E13, even for a small mitogen such as IGF2. Regardless, the assembly of the coronary vasculature during the same E10–13 period and its perfusion starting around E14 [15] represent a more efficient means of distributing growth-supporting factors to the full thickness of the myocardium throughout the rest of gestation and thereafter.

Our interest in the present study was to define the specific receptors which mediate IGF2 signaling in midgestation heart development. We demonstrate the primary role of IGF1R as the main receptor that transduces IGF2 signals in the developing myocardium. At present, we cannot explain why the INSR has no apparent genetic requirement and is activated (phosphorylated) at a much lower level than IGF1R in response to the same ligand, even when IGF1R is genetically removed from the myocardium. Certainly, in other contexts, the INSR responds robustly to IGF2 [10, 11]. One possibility is that INSR protein level is lower, although this is not apparent from the Western blot signal (Fig. 1a). Another possibility is that the INSR in embryonic myocardium is sequestered in a manner that limits its access to IGF2, for example in intracellular vesicles rather than at the cell surface. Certainly, other potential mechanisms might also explain the relative lack of INSR activity during the E10–13 period.

In our past [6, 7] and present analyses, we conditionally manipulated *Igf2* (ligand) and *Igf1r* and *Insr* (receptor) genes with *Nkx2.5Cre*, which is a knock-in of *Cre* sequence into the *Nkx2.5* gene [16]. It should be noted that this conditional approach circumvents the consequences when the *Igf2* and *Igf1r* genes are globally mutated, which both result in an overall embryo growth deficiency [17]; the conditional mutants thus demonstrate cardiac phenotypes in embryos of normal size and growth. In midgestation and later, the *Nkx2.5* gene is expressed only in cardiomyocytes, but in earlier development, it is expressed in the common progenitor from which the epicardium and myocardium both originate [18], thus explaining the relatively high efficiency of gene recombination that we observe in both lineages. For this reason, *Nkx2.5Cre* is a suitable driver line for both IGF2 ligand and IGF2 receptor manipulation. The epicardial source of IGF2 signals in heart development was confirmed by the equivalent ventricular phenotype when *Igf2* was conditionally ablated with *Nkx2.5Cre* or *Tbx18Cre*, but without phenotypic consequence when *Igf2* was conditionally ablated with *Tie2Cre* or *Myh6Cre* [7]. *Nkx2.5Cre* is active but not completely efficient in driving recombination in the endocardium, and most embryonic coronary vasculature originates from a source that does not express *Nkx2.5Cre* [13]. In the present analysis, while we see no evidence for any role for *Insr* in heart development when using *Nkx2.5Cre*, one caveat is the possibility that *Insr* may have a role in endocardium or coronary endothelium that escaped detection because of inefficient recombination. However, the similarity in phenotype between *Nkx2.5Cre/Igf2* and *Nkx2.5Cre/Igf1r* mutants makes this possibility remote, at least for INSR acting as an IGF2 receptor in the context of ventricular wall morphogenesis.

Zebrafish require IGF signaling for normal myocardial growth during heart development [19], illustrating that this pathway is conserved across vertebrate evolution. In zebrafish, the use of a small molecule antagonist specific for IGF1R indicated that this receptor mediates IGF signaling in the context of embryonic heart growth [19], just as we show here for mouse heart development. It is generally believed that similar mechanisms that occur during normal development are reiterated in the context of postnatal regeneration. In zebrafish, heart regeneration after adult injury occurs very efficiently, and adult zebrafish heart regeneration also requires IGF signaling [19, 20]. Mammalian hearts are regenerative after injury in the early neonatal period, but are mostly nonregenerative thereafter; the relative inability of the adult mammalian heart to regenerate underlies the high frequency of heart failure after adult myocardial infarction. In all contexts, the degree of heart regeneration is proportional to the degree of cardiomyocyte proliferation. Thus, the components that support mitogenic signaling in the normal embryonic heart, including IGF2 and its receptor IGF1R, may reveal pathways that are also relevant to mammalian postnatal cardiomyocyte proliferation and heart regeneration.

## Methods

### Mice

Conditional *Igf1r/Insr* mice [21], conditional *Igf2* mice [22], and *Nkx2.5Cre* [16] mice have been used in our previous studies [6], and were bred in-house for this project. All mice were on a mixed and unspecified strain background. Because the *Igf2* locus is subjected to imprinting [23], the conditional *Igf2* allele was always maintained in males and all experimental embryos were heterozygous for this paternally inherited allele. Pregnant adult female mice were anesthetized by isoflurane vapor inhalation prior to euthanasia by cervical dislocation.

### Morphometric heart analysis

E14.5 embryos were fixed overnight in 4% paraformaldehyde (PFA) in PBS then processed for paraffin embedding. Serial sections were taken and stained with hematoxylin and eosin. Transverse sections at the level of the aortic valve were used for measurement of ventricular wall thickness at a position 45° clockwise (right ventricle) or counterclockwise (left ventricle) to the ventricular septum–apex axis. Image J was used to measure the ventricular wall thickness. Statistical comparisons were made using an unpaired T test.

### Immunofluorescence (IF)

E11.5, E12.5 and E14.5 embryos were fixed with 4% PFA in PBS at 4 °C overnight. After cryoprotection in 10 and 30% sucrose, the tissue was embedded and frozen in OCT. Eight micrometer sections were used for IF. Slides were briefly fixed with 4% PFA, washed and permeabilized with PBS containing 0.1% Triton X-100 at room temperature for three times, 5 min each. After blocking with 10% normal donkey serum and 1% BSA at room temperature for 1 h, sections were incubated in rabbit anti-p-IGF-IR (Santa Cruz SC101703) and goat anti-Troponin C (Abcam ab30807), or goat anti-p-Insulin receptor (pINSR) (SC25103) and rabbit anti- MYL2 (Abcam ab79935) in PBS containing 1% BSA and 10% donkey serum at 4 °C overnight. Secondary antibodies donkey anti-rabbit (Invitrogen Alexa Fluor 546) and donkey anti-goat (Invitrogen Alexa Fluor 488), or donkey anti-goat (Invitrogen Alexa Fluor 546) and donkey anti-rabbit (Invitrogen Alexa Fluor 488) were used to detect pIGF1R and Troponin C, or pINSR and MYL2 with 1 h incubation at room temperature. Slides were mounted with mounting media containing DAPI.

### Polymerase chain reaction (PCR)

RNA was extracted from heart ventricle by using Quick-RNA Mini Prep Kit (Zymo research). Equal amounts of RNA were used to synthesize cDNA using MMLV reverse transcriptase (Invitrogen). The following primer sequences were used to amplify *Insr* cDNA fragments to detect splicing: 5′- GGTGTACTGGGAGAGGCAAG -3′ and 5′- CGGTACCCAGTGAAGTGTCT -3′. Beta-actin was used as the internal control.

### Western blot

Proteins separated on SDS-PAGE gels were transferred to polyvinylidene difluoride membranes (Biorad). Membranes were blocked in PBS-Tween with 5% nonfat dry milk. Primary antibodies for detection of IGF1R (Cell Signaling 9750) and INSR (Santa Cruz SC711) were used at 1:1000 dilution. Bound primary antibodies were visualized using secondary antibodies conjugated to horseradish peroxidase (1:3000, Santa Cruz Biotechnology) and chemiluminescent substrate (ECL plus, Thermo Scientific).

## Additional files

Additional file 1: **Figure S1.** Activated receptors are in cardiomyocytes. High magnification confocal visualization of activated (phosphorylated) receptors in the ventricular wall of an E12.5 control heart indicates that staining occurs in cardiomyocytes. Image presentation (single channel and merged channels) and abbreviations are as in the legend to Fig. 2. Scale bar: 20 μm. (PDF 633 kb)

Additional file 2: Raw data of ventricular wall thickness in E14.5 embryos. Primary raw data graphed in Fig. 3 are provided in this Additional File. (XLSX 13 kb)

## Abbreviations

DAPI: 4′,6-diamidino-2-phenylindole
E: Embryonic day
IF: Immunofluorescence
IGF1R: Insulin-like growth factor 1 receptor
IGF2: Insulin-like growth factor 2
INSR: Insulin receptor
PCR: Polymerase chain reaction
PFA: Paraformaldehyde
