# Supplementary figures and images for: Differential roles of insulin like growth factor 1 receptor and insulin receptor during embryonic heart development
Source: BMC Dev Biol. 2019 Mar 25;19:5. doi: 10.1186/s12861-019-0186-8 (PMC6434851; doi:10.1186/s12861-019-0186-8)

Supplemental Figure 1

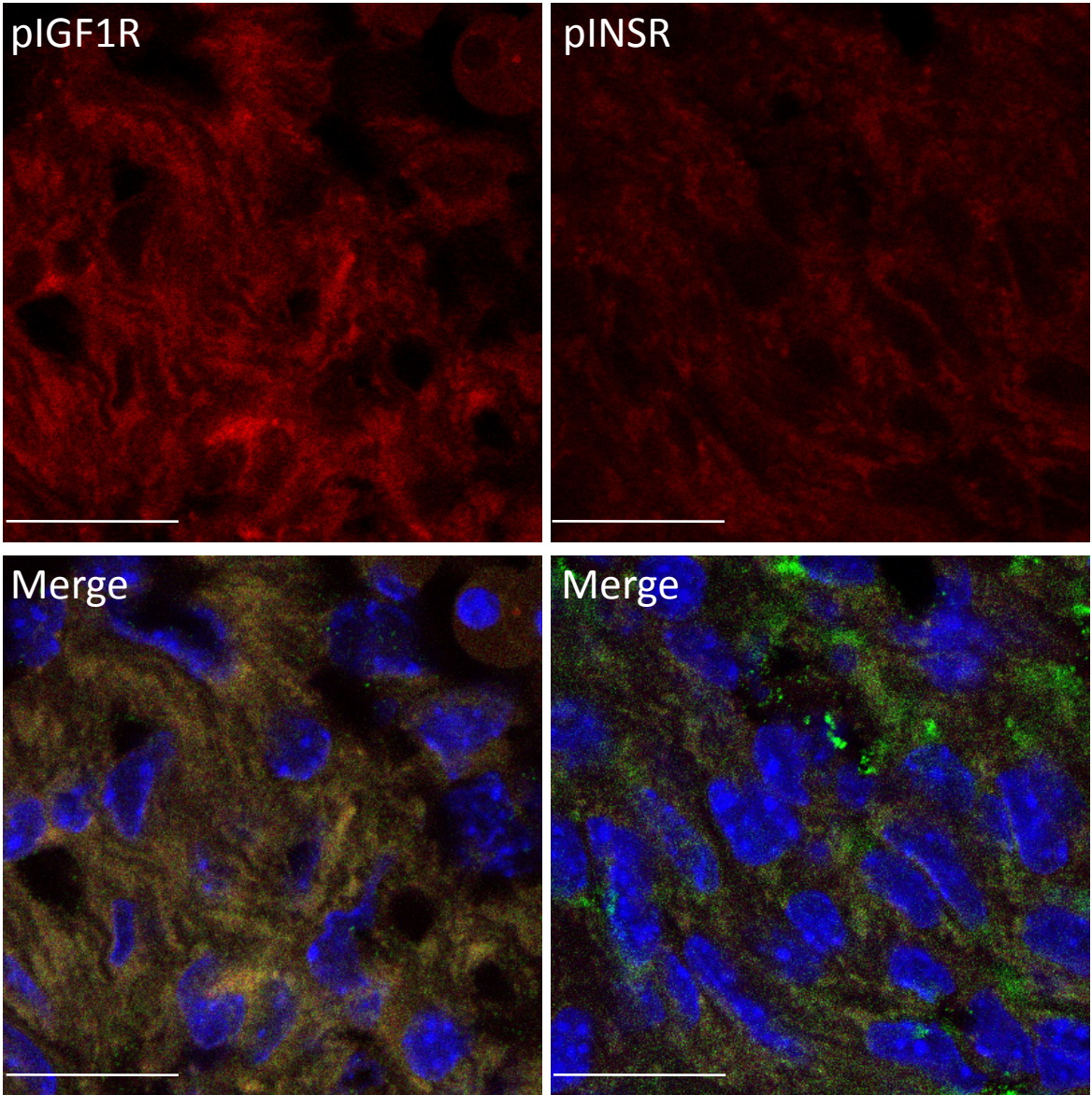

Supplement: Supplementary file 1 — Figure S1. Activated receptors are in cardiomyocytes. High magnification confocal visualization of activated (phosphorylated) receptors in the ventricular wall of an E12.5 control heart indicates that staining occurs in cardiomyocytes. Image presentation (single channel and merged channels) and abbreviations are as in the legend to Fig. 2. Scale bar: 20 μm. (PDF 633 kb) [file 12861_2019_186_MOESM1_ESM.pdf]
